# Editorial: Oxylipins: The Front Line of Plant Interactions

**DOI:** 10.3389/fpls.2022.878765

**Published:** 2022-03-28

**Authors:** Koichi Sugimoto, Silke Allmann, Michael V. Kolomiets

**Affiliations:** ^1^Tsukuba-Plant Innovation Research Center, University of Tsukuba, Ibaraki, Japan; ^2^Department of Plant Physiology, Green Life Sciences Research Cluster, Swammerdam Institute for Life Sciences, University of Amsterdam, Amsterdam, Netherlands; ^3^Department of Plant Pathology and Microbiology, Texas A&M University, College Station, TX, United States

**Keywords:** plant oxylipins, oxidized lipids, volatile organic compounds, biotic interactions, abiotic stresses, jasmonates

In this Research Topic, a collection of high-quality papers represents advances in our understanding of synthesis, biological functions and signaling activities of volatile and non-volatile plant oxylipins. Oxylipins are a large group of functionally and structurally diverse molecules produced from oxidized lipids and are widely known to have various roles in plant growth, development and interactions with biotic and abiotic stressors. Here we introduce 10 original research papers and two review papers on plant-environment, plant-pathogen, and plant-plant interactions. In this editorial piece, we overview the oxylipin metabolic pathways and introduce each research contribution in accordance with the corresponding metabolic pathway branches depicted in Figure 1.

**Figure 1:**
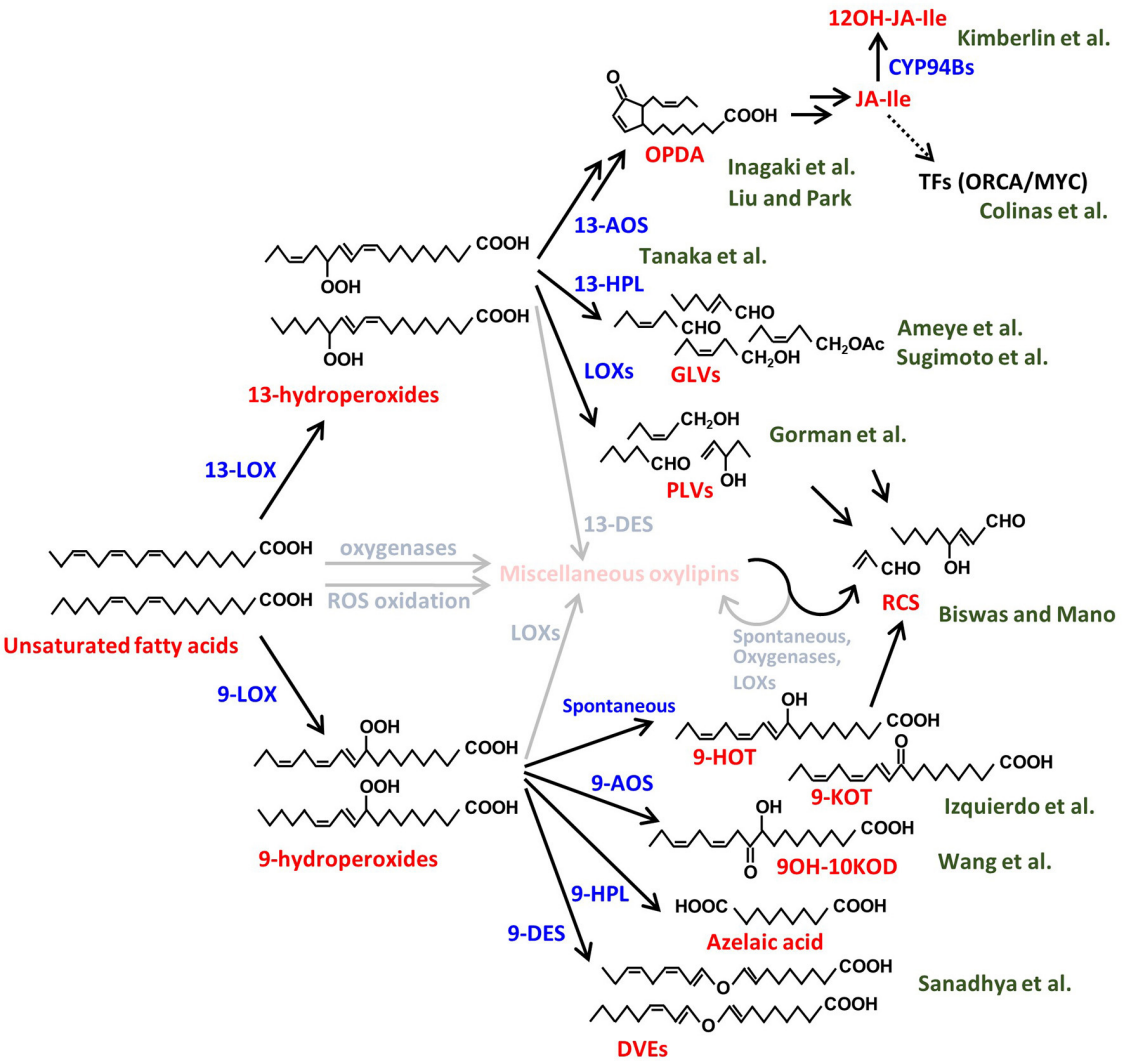
Biosynthetic pathways of various plant oxylipins. Biosynthesis of the plant oxylipins is initiated by oxidation of the unsaturated fatty acids, e.g., linoleic acid and linolenic acid in this figure, enzymatically or nonenzymatically. The compound names are shown in red and the processes are shown in blue. The green characters behind the compounds show the author names in this Research Topic and the relationships between the authors' papers and the compounds. The miscellaneous compounds and processes that are not described well in this Research Topic are shown in faded colors. LOX, lipoxygenase; AOS, allene-oxide synthase; HPL, hydroperoxide lyase; DES, divinyl ether synthase; CYP, cytochrome P450; ROS, reactive oxygen species; OPDA, 12-oxo-phytodienoic acid; JA-Ile, jasmonic acid-isoleucine conjugate; 12OH-JA-Ile, 12-hydroxy JA-Ile; TFs, transcription factors; GLVs, green leaf volatiles; PLVs, pentyl leaf volatiles; RCS, reactive carbonyl species; 9-HOT, 9-hydroxy octadecatrienoic acid; 9-KOT, 9-keto-octadecatrienoic acid; 9OH-10KOD, 9-hydroxy-10keto-octadecadienoic acid; DVEs, divinyl ethers.

Jasmonates (JAs) are the best-studied group of plant oxylipins and are widely known as defense phytohormones. JA biosynthesis is triggered by the reaction of 13-lipoxygenase (13-LOX) on polyunsaturated fatty acids, which are abundant in plant membrane lipids, to produce 13-hydroperoxides (Figure 1). 13-Hydroperoxides are catalyzed by 13-allene oxide synthase (13-AOS), a member of the CYP74 family, and subsequently converted into 12-oxo-phytodienoic acid (OPDA) and JA by the sequential reactions of allene oxide cyclase, OPDA reductase, and β-oxidation processes (Acosta and Farmer, 2010). The major bioactive JA in vascular plants is the JA-Isoleucine conjugate (JA-Ile), which is recognized by the COI1 receptor complex (Yan et al., 2013). The JA signal activates a group of downstream transcription factors responsible for the transcriptional upregulation of defense genes (Yan et al., 2013). One of the common defense responses in plants is the activation of secondary metabolite accumulation, such as alkaloids and terpenoids, which act as toxic compounds to pathogens and pests (Ding et al., 2021). Many drugs used in modern medicine are derived from such plant secondary metabolites. For example, *Catharanthus roseus* is known to produce the anticancer alkaloids vinblastine and vincristine, belonging to the class of monoterpenoid indole alkaloids. Colinas et al. combined newly generated and publicly available transcriptome data of *Catharanthus roseus* to identify new members of the BIS and ORCA transcription factors that specifically regulate genes involved in the synthesis of these alkaloids.

Although JA signaling strongly induces defense responses, such activation is tightly controlled not to impede plant growth while defending (Guo et al., 2018). To prevent excessive induction of the defense, the active form of JA is rapidly converted to the inactive forms through hydroxylation and/or other metabolic processes (Koo et al., 2011). Kimberlin et al. report novel translational regulation of proper JA responses through the analysis of an Arabidopsis double mutant of cytochrome P450s, CYP94B1 and CYP94B3, which convert active JA-Ile to 12OH-JA-Ile. Bioactive JA molecules in plants are not only JA-Ile but also its biosynthetic intermediate, OPDA. Liu and Park reviewed recent studies uncovering the biological functions of OPDA, especially in terms of growth-defense trade-offs and environmental responses in vascular plants. Unlike vascular plants, mosses do not produce JA or JA-Ile in response to mechanical damage but accumulate its cyclopentenone precursor compounds OPDA and dinor-OPDA, the latter of which is synthesized from hexadecatrienoic acid rather than linolenic acid (Yan et al., 2013). These compounds have been shown to function through both COI1-dependent and -independent signaling mechanisms in mosses (Monte et al., 2018, 2020). Inagaki et al. identified through transcriptome- and genome-mining the jasmonate signaling components, COI1 receptor, JAZ repressor, and MYC transcription factor, in the moss species, *Calohypnum plumiforme*, and suggested that OPDA is involved in primitive defense signaling, most likely via the COI1-dependent signaling pathway.

In addition to the 13-AOS-mediated JA biosynthesis, plants convert 13-hydroperoxides into green leaf volatiles (GLVs) and divinyl ethers (DVEs) by other CYP74 family proteins, 13-hydroperoxide lyase (13-HPL) and 13-divinyl ether synthase (13-DES), respectively (Matsui, 2006). GLVs are responsible for the leafy-green scent produced immediately after leaf damage, and the rapid production of GLVs, called GLV-burst, is commonly observed in vascular plants (e.g., D'Auria et al., 2007). Tanaka et al. studied whether the GLV-burst is a trait that has been conserved from primitive plants by comparing a wide range of plants and found that the GLV-burst occurs in all fern species, while being absent in most of the moss species. One of the well-studied biological functions of GLVs is the airborne communication between plants and surrounding organisms (Matsui, 2006; Scala et al., 2013). Ameye et al. utilized an untargeted metabolomics approach to investigate the effect of GLV, *Z*-3-hexenyl acetate, on the activation of wheat responses to infection by *Fusarium graminearum*, a major mycotoxin producing pathogen of wheat. Among the major metabolic changes identified were induction of ROS followed by production of diverse phenylpropanoids with antioxidative activities accompanied by a strong induction of glycosylation processes. Sugimoto et al. showed that various airborne GLVs, aldehydes, alcohols, and esters, could be glycosylated in tomato and Arabidopsis leaves through endogenous metabolic processes using mutants of GLV biosynthetic genes. Plants emit not only GLVs, with a six-carbon backbone, but also pentyl leaf volatiles (PLVs), which have a five-carbon backbone. Gorman et al. clarified the order of biosynthesis of diverse PLVs in Arabidopsis and maize and demonstrated that, in sharp contrast to the virulence-promoting effect of GLVs on the anthracnose pathogen, *Colletotrichum graminicola*, PLV treatment of maize increased resistance to this pathogen by activation of production of oxylipin ketols.

In addition to the 13-LOX-initiated pathways, plants have another oxylipin pathway initiated by 9-LOX to produce 9-hydroperoxides (Liavonchanka and Feussner, 2006). Similar to the 13-oxylipin pathways, 9-LOXs initiate synthesis of various 9-oxylipins by the reaction of 9-AOS, 9-HPL, 9-DES, and other unidentified enzymes (Figure 1). Sanadhya et al. reported that the biosynthesis of tomato DVEs, colneleic acid and colnelenic acid, produced by 9-DES are modulated by infection of root-knot nematode, *Meloidogyne javanica*. 9-DES expression is associated with the nematode feeding site and is modulated by auxin and salicylic acid and its overexpression results in increased resistance. Evidence is presented that DVEs have two modes of action, as they decrease motility of nematodes and act as defense signals. Wang et al. presented the physiological function of miscellaneous 9-oxylipins using 9-LOX mutants. The 9-LOX mutant of maize displayed increased susceptibility to Gibberella stalk rot caused by *Fusarium graminearum*, indicating that 9-oxylipins contribute to resistance to this pathogen. The transcriptome analysis and oxylipin profiling revealed that 9-oxylipin and JA pathway are antagonistically regulated and points to the previously unrecognized role of JA in promoting susceptibility to this hemi-biotrophic pathogen. On the other hand, Izquierdo et al. applied a different approach, forward genetic screening of a series of noxy (non-sensitive to oxylipin) mutants of mitochondrial proteins, which are insensitive to exogenous 9-HOT/9-KOT to clarify the role of 9-oxylipins. The authors clarified the oxidative stress targets of the 9-oxylipin signaling by showing that 9-oxylipins reduce respiration inhibitor-induced oxidative stress by activation of a specific mitochondria retrograde signaling pathway. Moreover, the authors showed that mitochondrial complex III is a target of the signaling activity of both 9- and 13-oxylipin pathways with α,β-unsaturated structure during oxidative stress responses.

As described above, plant oxylipins are produced by oxygenation of polyunsaturated fatty acids and downstream metabolic enzymes. In addition to the enzymes focused on in this Research Topic's papers, there are various oxylipin biosynthetic enzymes, such as those initiated by dioxygenases. Moreover, non-enzymatic oxidation by reactive oxygen species and subsequent cleavage/conversion of oxidized lipids also drives the production of a wide variety of oxylipins. Biswas and Mano categorized a series of compounds, which have α,β-unsaturated carbonyl structures, as reactive carbonyl species and reviewed their reaction targets and physiological functions. The functions of most oxylipins are still unclear. Although physiological roles of some of them are suggested, their mode of action is still not fully understood.

In conclusion, the papers collected in this Research Topic have presented some of the recent advances in oxylipin biology and will be informative for the researchers in this area and also help attract additional researchers to this growing research area of plant oxylipins.

## Author Contributions

All authors listed have made a substantial, direct, and intellectual contribution to the work and approved it for publication.

## Funding

This work was supported by JSPS KAKENHI Grant Number JP20H03278 to KS, USDA-NIFA grant awards 2017-67013-26524 and 2021-67013-33568 to MK, and European Research Council (ERC) under the European Union's Horizon 2020 research and innovation programme (grant agreement no. 805074) to SA.

## Conflict of Interest

The authors declare that the research was conducted in the absence of any commercial or financial relationships that could be construed as a potential conflict of interest.

## Publisher's Note

All claims expressed in this article are solely those of the authors and do not necessarily represent those of their affiliated organizations, or those of the publisher, the editors and the reviewers. Any product that may be evaluated in this article, or claim that may be made by its manufacturer, is not guaranteed or endorsed by the publisher.
